# Central nervous system gadolinium accumulation in patients undergoing periodical contrast MRI screening for hereditary tumor syndromes

**DOI:** 10.1186/s13053-017-0084-7

**Published:** 2018-01-05

**Authors:** Evelynn Vergauwen, Anne-Marie Vanbinst, Carola Brussaard, Peter Janssens, Dieter De Clerck, Michel Van Lint, Anne C. Houtman, Olaf Michel, Kathelijn Keymolen, Bieke Lefevere, Susanne Bohler, Dirk Michielsen, Anna C. Jansen, Vera Van Velthoven, Sven Gläsker

**Affiliations:** 1 Department of Neurosurgery, Universitair Ziekenhuis Brussel, Laarbeeklaan 101, 1090 Brussels, Belgium; 2 Department of Radiology, Universitair Ziekenhuis Brussel, Brussels, Belgium; 3 Department of Nephrology, Universitair Ziekenhuis Brussel, Brussels, Belgium; 4 Department of Ophthalmology, Universitair Ziekenhuis Brussel, Brussels, Belgium; 5 Department of Otorhinolaryngology, Universitair Ziekenhuis Brussel, Brussels, Belgium; 6 Department of Genetics, Universitair Ziekenhuis Brussel, Brussels, Belgium; 7 Department of Psychology, Universitair Ziekenhuis Brussel, Brussels, Belgium; 8 Department of Urology, Universitair Ziekenhuis Brussel, Brussels, Belgium; 9 Department of Pediatrics, Universitair Ziekenhuis Brussel, Brussels, Belgium

**Keywords:** Von Hippel-Lindau disease, Tuberous Sclerosis Complex, Gadolinium accumulation, MRI screening, Familial tumor syndromes

## Abstract

**Background:**

Patients with hereditary tumor syndromes undergo periodical magnetic resonance imaging (MRI) screening with Gadolinium contrast. Gadolinium accumulation has recently been described in the central nervous system after repeated administrations. The prevalence and rate of accumulation in different subgroups of patients are unknown. Neither are the mechanism nor clinical impact. This may cause uncertainty about the screening. To explore the prevalence and rate of Gadolinium accumulation in different subgroups, we retrospectively analyzed MRIs of patients with von Hippel-Lindau disease (VHL) and Tuberous Sclerosis Complex (TSC).

**Methods:**

We determined the prevalence and rate of accumulation in the dentate nucleus and globus pallidus on unenhanced T1-weighted MRI from VHL and TSC patients. We compared the signal intensities of these regions to the signal intensity of the pons. We evaluated the impact of number of MRIs, kidney function and liver function on Gadolinium accumulation.

**Results:**

Twenty eight VHL patients and 24 TSC patients were included. The prevalence of accumulation in the dentate nucleus and globus pallidus increased linearly according to number of Gadolinium enhanced MRIs and was higher in the VHL group (100%). A significant linear correlation between number of MRIs and increased signal intensity was observed in the VHL group.

**Conclusions:**

Gadolinium accumulation occurs in almost all patients undergoing contrast MRI screening after >5 MRIs. We advocate a screening protocol for patients with hereditary tumor syndromes that minimizes the Gadolinium dose. This can be accomplished by using a single administration to simultaneously screen for brain, spine and/or abdominal lesions, using an MRI protocol focused on either VHL- or TSC-specific lesions. Higher prevalence and rate of accumulation in VHL patients may be explained by the typical vascular leakage accompanying central nervous system hemangioblastomas.

## Background

Patients with hereditary tumor syndromes need to undergo periodical magnetic resonance imaging (MRI) screening, according to international guidelines [1, 2] Therefore, these patients are repeatedly exposed to intravenous Gadolinium (Gd) based contrast agents (GBCAs). After the Food and Drug Administration published a Public Health Advisory (2006) on GBCAs and the associated risk of nephrogenic systemic sclerosis, the use of GBCAs has been globally reduced, especially in patients with severe renal impairment. The association with nephrogenic systemic sclerosis is markedly stronger for *high risk* linear GBCAs than for *low risk* macrocyclic GBCAs [3–5].

Gd accumulation has been described in the skin, bone, liver, kidney, muscles and spleen.

Recently, Kanda et al. [6] reported Gd accumulation in the central nervous system, more specifically in the dentate nucleus and globus pallidus, in patients who underwent at least 5 MRI scans with intravenous linear GBCAs. They observed a cumulative dose-dependent relationship, even in patients with a normal renal function. Accumulation has now been described for both linear and macrocyclic GBCAs [7].

To date, the potential clinical impact of Gd accumulation remains to be demonstrated [8, 9]. Several case reports have been published by different groups, including Maramattom et al., Hui et al. and Naganawa et al., describing side-effects such as decreased consciousness, hemiparesis and encephalopathy following intravenous Gd administration [10–12].

A Gadolinium-toxicity support group was recently established. The group has published a paper in which symptoms such as chronic pain, worsening vision, dermal changes, tinnitus and balance problems are reported 1 month after Gd administration. However, this paper is rather a self-report of patients than a controlled study [13, 14]. Neither large series nor long term prospective studies have been performed in humans. Preclinical safety animal studies on intravenous Gd administration have only demonstrated neurological side-effects in rats with a disrupted blood-brain barrier [15]. Whether accumulation may lead to long term neurological consequences remains unknown, but the liberal use of all GBCAs in the context of regular screening regimens should be reevaluated.

It is not known whether Gd accumulation may be influenced by the type of underlying disease. Knowledge about this subject is limited. For example, some researchers have put forward that the intensity of the dentate nucleus in multiple sclerosis patients is more influenced by disease progression, than by Gd accumulation. Second, others believe that the proximity of a tumor to the dentate nucleus may catalyze accumulation. Third, some have suggested that children may be more susceptible for accumulation compared to adults. Additionally, independent studies report inconsistent results about the quantity of Gd accumulation with identical contrast agents. Together, these differences may be explained by various host factors [16]. Especially the prevalence and speed of accumulation in different subgroups of patients with hereditary tumor syndromes has thus far not been evaluated and may be explanatory for individual differences in accumulation.

In a recent large systematic review, Gulani et al. have suggested priorities for future research about Gd accumulation. They emphasized that the prevalence in different subgroups, rate, mechanism and clinical impact of Gd accumulation in the central nervous system require further investigation [7, 16].

Our hospital serves as a tertiary reference center for patients with von Hippel-Lindau disease (VHL) and Tuberous Sclerosis Complex (TSC), both autosomal dominant hereditary tumor syndromes. In VHL, a mutation in the VHL-gene leads to a predisposition for cerebellar, spinal and retinal hemangioblastomas, renal cysts and renal cell carcinoma (RCC), pheochromocytomas and endolymphatic sac tumors [17]. In TSC, a mutation in either the TSC1 or TSC2 gene predisposes for the formation of hamartomas (benign tumors) in the central nervous system, skin, kidneys, heart, eyes and other organs. Rarely, RCC occurs [18]. Consequently, these patients are exposed to an average of 1 Gd dose per year for MRI screening of the brain, abdomen and/or spine. We retrospectively analyzed the prevalence and rate of accumulation in a consecutive series of these patients with VHL and TSC. Linkage of disease pathophysiology to speed of Gd accumulation may reveal new mechanisms of accumulation.

## Methods

In a consecutive series of patients with VHL and TSC, we retrospectively evaluated the prevalence and rate of Gd accumulation in the dentate nucleus and globus pallidus on unenhanced T1-weighted (T1-w) MRI. Retrospectively, the signal intensity of the dentate nucleus and globus pallidus was compared to the number of Gd enhanced MRIs. We performed both qualitative (prevalence of accumulation) and quantitative analyses (amount of accumulation), as recently suggested by Ramalho et al. [19]. Furthermore, we evaluated kidney and liver function as potential contributing factors for Gd accumulation. We believe that the evaluation of kidney function is especially relevant in this setting, because some VHL and TSC patients have lost some of their kidney function due to renal cell carcinoma and angiomyolipomas, respectively.

### Patient selection and data collection

We screened the database of all VHL and TSC patients who are regularly followed for their disease in our hospital. Inclusion criteria were: (1) diagnosis of VHL or TSC based on either clinical and/or genetic criteria, according to the guidelines [1, 20] (2) recent unenhanced T1-w MRIs of the brain available and (3) date of diagnosis and therefore duration of screening known.

Some TSC patients had previously underwent MRIs in different institutions. Consequently, the exact number of MRIs could not always be deducted from their electronic files. However, based on the guidelines, VHL and TSC patients undergo an average of 1 Gd enhanced MRI per year of screening. Therefore we opted to use duration of screening, instead of the amount of MRIs in the patients’ files, as a parameter to determine ‘number of MRIs’ in both VHL and TSC groups. Duration of screening itself was calculated as date of data analysis (11 April 2017) minus date of diagnosis. As the diagnosis of the majority of VHL patients was made in our own institution, the exact number of MRIs was known for most of them and the calculation (1 MRI per year) almost exactly corresponded to the actual amount of MRIs as mentioned in the electronic files.

Patients with sporadic hemangioblastomas were excluded. Sex and age were recorded. The patients’ electronic files were screened for immediate and long term clinical signs of intravenous Gd toxicity.

### Kidney and liver function

Kidney and liver function as potential contributing factors for accumulation of Gd were evaluated. The most recent available laboratory data were obtained. Kidney function was considered to be normal when the estimated Glomerular Filtration Rate (eGFR) was above 90 ml/min/1.73m^2^ (MDRD formula, Modification of Diet in Renal disease). Liver function was assessed using values of blood albumin (g/l), prothrombin time (INR, International Normalized Ratio) and total bilirubin (μmol/l) and considered to be normal when all three values were between reference ranges. The reference ranges for blood albumin were 32–54 g/l for 5–19 years (age), 35–52 g/l for 20–59 years and 32–46 g/l for 60–90 years. The reference range for prothrombin time was 0.9–1.20 INR and for total bilirubin 5.08–20.31 μmol/l.

### Imaging data

For every patient, the last available unenhanced T1-w MRI of the brain was observed in the axial, coronal and sagittal plane. All images were analyzed using Impax Viewer (Agfa Health Care). The images were interpreted by two members of the department of neurosurgery (one resident and one senior neurosurgeon) and one radiologist with >20 years of experience, of whom the latter was blinded to *years of screening*.

As patients underwent MRI scanning in different hospitals and countries, the properties of the MRI scanners (e.g. repetition time, echo time, section thickness, field of view, inversion recovery delay and in plane resolution) differed. The same issue was encountered for the dose of GBCAs, which was not always known. By contacting the hospitals where the MRIs were performed, it was clear that patients had especially received linear GBCAs: 10–15 ml gadopentetate dimeglumine (Magnevist ®, 0.5 mmol/ml solution), 10–15 ml gadodiamide (Omniscan ®, 0.05 mmol/ml solution) or 10–15 ml gadobenate dimeglumine (Multihance ®, 0.05 mmol/ml solution). Since April 2016, patients had also received 10–15 ml gadoterate meglumine (Dotarem ®, 0.05 mmol/ml solution), a macrocylic GBCA used in our center.

Some of the included patients with VHL disease suffered from large hemangioblastomas that occupied part of the cerebellum. Other patients had already underwent extended cerebellar resections. As a consequence, both dentate nucleus and globus pallidus regions could not always be visualized simultaneously. Therefore, spontaneous hypersignal of the dentate nucleus and spontaneous hypersignal of the globus pallidus were evaluated in two separate analyses.

First, the prevalence of hypersignal in the dentate nucleus and globus pallidus was evaluated by subjective visualization in both groups. The dentate nucleus is typically not visible on unenhanced T1-w MRI and the globus pallidus is typically hypointense. Visible hyperintensity of these regions on unenhanced T1-w MRI was scored as *present* or *absent*.

Second, the objective signal intensity of the dentate nucleus and globus pallidus was compared to the signal intensity of the pons. The pons was chosen as area of normalization, because it is the area where the least Gd accumulation occurs [21]. Five regions of interest (ROI) were drawn per patient: left dentate nucleus, right dentate nucleus, left globus pallidus, right globus pallidus and pons. Placement of ROIs on unenhanced T1-w MRIs was guided by identification of the dentate nucleus and globus pallidus on unenhanced T2-w MRIs. The result was expressed as signal intensity ratio (signal intensity dentate nucleus/signal intensity pons left and right, signal intensity globus pallidus/signal intensity pons left and right) and compared to number of MRIs, in order to analyze the rate by which Gd accumulation occurs.

The prevalence of hypersignal and the signal intensity ratios of the VHL and TSC groups were compared to each other.

### Statistical analysis

Statistical analysis was conducted using SPSS software (Statistical Package for the Social Science, IBM analytics, version 24). The a priori significance level was set at *p* < 0.05. A bivariate Pearson’s correlation test was used to assess if a statistically significant correlation existed between number of Gd enhanced MRIs and signal intensity ratio (signal intensity dentate nucleus/pons left and right, signal intensity globus pallidus/pons left and right). In order to quantify a potential relationship between number of Gd enhanced MRIs and signal intensity ratios, a linear regression model was used.

## Results

### Patient characteristics

Of 33 patients followed for VHL in our center, 28 met the inclusion criteria. Five patients were excluded due to insufficient imaging material. VHL disease was diagnosed clinically in 11 patients and both clinically and genetically in 17 patients. The patients were screened between 1987 and 2017. They were allocated to groups according to number of Gd enhanced MRIs in the past: 0 to 5, 6 to 10, 11 to 15, 16 to 20 or >20 Gd enhanced MRIs.

Of 25 patients that are regularly followed for TSC, 24 met the inclusion criteria. One patient was excluded due to insufficient imaging material. TSC was diagnosed clinically in 5 patients and both clinically and genetically in 19 patients. The patients were screened between 1987 and 2017 and also allocated to groups according to number of Gd enhanced MRIs in the past.

In our retrospective analysis we could not find any documented clinical signs of toxicity immediately after intravenous Gd administration, nor was there any mention of inexplicable symptoms in the patients’ files that could not be related to VHL or TSC disease progression.

The characteristics of each group are summarized in Table 1.

**Table 1:** Patient characteristics of VHL and TSC groups

Parameter	VHL group	TSC group
Patients included	28	24
Clinical signs of Gadolinium toxicity	0	0
Radiological signs of Gadolinium accumulation	15	10
Mean age (y)	39 (16 to 63)	27 (4 to 67)
Mean number of MRIs (y)	10 (0 to >20)	16 (2 to >20)
Patient sex		
Men	11	11
Women	17	13
Kidney disease		
No kidney disease	9	3
Few bilateral cysts	10	5
Multiple cysts	2	3
Renal cell carcinoma	7	1
Angiomyolipomas	/	12
(Ab)normal kidney function		
Stage 1 (eGFR normal >90 ml/min/1.73m^2^)	25	20
Gadolinium accumulation	12/25	13/20
Stage 2 (eGFR 90–60 ml/min/1.73m^2^)	3	1
Gadolinium accumulation	2/3	1/1
Stage 3 (eGFR 30–59 ml/min/1.73m^2^)	1	3
Gadolinium accumulation	1/1	2/3
Abnormal liver function	0	0

Note: data are number of patients, unless indicated otherwise

All patients with AML also had few or multiple bilateral cysts, except for 1 patient with only AML

### Gadolinium accumulation: Prevalence

**Fig. 1:**
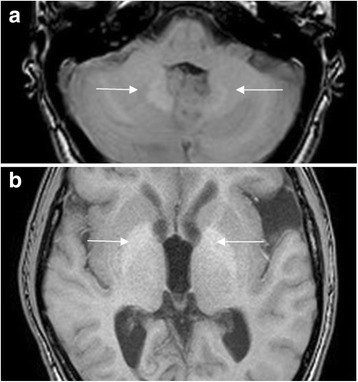
Unenhanced T1-w MRI of a VHL patient, showing spontaneous hypersignal (arrows) in dentate nucleus (**a**) en globus pallidus (**b**)

**Fig. 2:**
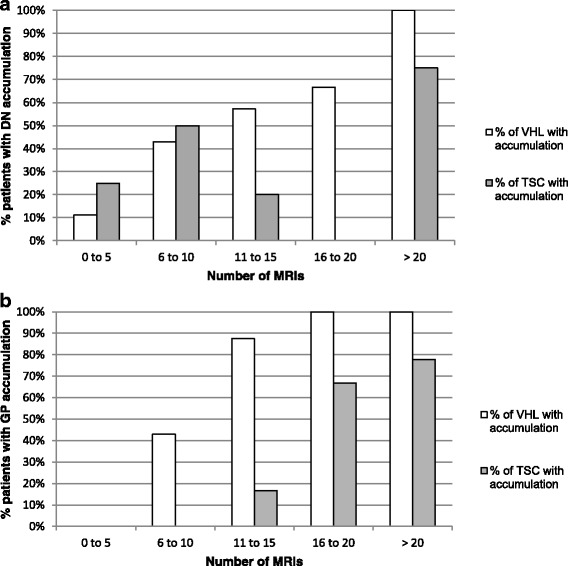
Different dynamics of Gd accumulation in dentate nucleus (DN) (**a**) and globus pallidus (GP) (**b**), in VHL and TSC groups. In both groups, the prevalence of patients with spontaneous hypersignal on unenhanced T1-w MRI in the DN and GP increased linearly with the number of Gd enhanced MRIs. The increase was highest in the VHL group

In 27 of 28 included VHL patients, the region of the dentate nucleus could be identified on unenhanced T1-w MRI of the brain. One patient had undergone a large cerebellar resection and therefore the region of the dentate nucleus could not be visualized. There was a significant positive correlation between the number of Gd enhanced MRIs and the prevalence (in %) of patients with hypersignal in the dentate nucleus (11% in the group of 0 to 5 MRIs, 43% in the group of 6 to 10 MRIs and 57 to 100% in the groups of 11 to more than 20 MRIs). (Fig. 1a, Fig. 2 and Table 2).

**Table 2:** Prevalence of Gadolinium accumulation in dentate nucleus and globus pallidus; in VHL and TSC groups

Number of MRIs	Dentate nucleus			Globus pallidus		
	accumulation	no accumulation	% total*	accumulation	no accumulation	% total*
VHL group						
0 to 5	1/9	8/9	11.11%	0/8	8/8	0.00%
6 to 10	3/7	4/7	42.86%	3/7	4/7	42.86%
11 to 15	4/7	3/7	57.14%	7/8	1/8	87.50%
16 to 20	2/3	1/3	66.67%	3/3	0/3	100.00%
> 20	1/1	0/1	100.00%	2/2	0/2	100.00%
	total: 27			total: 28		
TSC group						
0 to 5	1/4	3/4	25.00%	0/4	4/4	0.00%
6 to 10	1/2	1/2	50.00%	0/2	2/2	0.00%
11 to 15	1/5	4/5	20.00%	1/6	5/6	16.67%
16 to 20	0/2	2/2	0.00%	2/3	1/3	66.67%
> 20	6/8	2/8	75.00%	7/9	2/9	77.78%
	total: 21			total: 24		

In both groups, the prevalence of patients with hypersignal in the dentate nucleus and globus pallidus increased linearly with the number of Gd enhanced MRIs. The increase was highest in the VHL group

*% total = % of patients with accumulation

Note: data are number of patients, unless indicated otherwise

The globus pallidus could be identified in all 28 included VHL patients. The prevalence of patients with globus pallidus hypersignal also increased linearly according to increasing numbers of Gd enhanced MRIs (0% in the group of 0 to 5 MRIs, 43% in the group of 6 to 10 MRIs and 87 to 100% in the groups of 11 to more than 20 MRIs). (Fig. 1b, Fig. 2 and Table 2).

In the group of TSC patients, the dentate nucleus region and globus pallidus could be identified in 21 of 24 and all 24 patients respectively. In 3 patients the dentate nucleus region could not be visualized due to artifacts or insufficient spatial resolution of the images. A significant linear increase in prevalence of hypersignal was observed (for both regions: 25% in the group of 0 to 5 MRIs, 50% in the group of 6 to 10 MRIs and 78% in the groups of 11 to more than 20 MRIs). (Fig. 2 and Table 2).

### Gadolinium accumulation: Signal intensity ratios (signal intensity dentate nucleus/pons and signal intensity globus pallidus/pons)

In both groups (VHL and TSC), a linear correlation was observed between the number of Gd enhanced MRIs and increase in signal intensity ratio in the dentate nucleus and globus pallidus. Although the linear correlation was present and statistically significant in the VHL group (*R* = 0.472–0.633 and p = <0.001–0.013), it was present but not statistically significant in the TSC group (*R* = 0.368–0.447 and *p* = 0.029–0.101). (Table 3).

**Table 3:** Results of correlation and linear regression analysis

Number of MRIs	Regression Coefficient	95% CI	Standardized Regression Coefficient	Significance
VHL				
vs. SI Ratio right DN/pons	0.004	0,001–0,008	0.472	0.013
vs. SI Ratio left DN/pons	0.005	0,001–0,008	0.487	0.01
vs. SI Ratio right GP/pons	0.008	0,003–0,013	0.563	0.002
vs. SI Ratio left GP/pons	0.009	0,004–0,013	0.633	<0,001
TSC				
vs. SI Ratio right DN/pons	0.002	0,000–0,004	0.402	0.071
vs. SI Ratio left DN/pons	0.002	−0.006	0.368	0.101
vs. SI Ratio right GP/pons	0.003	0,000–0,007	0.385	0.063
vs. SI Ratio left GP/pons	0.004	0,000–0,008	0.447	0.029

In both groups, a linear correlation was seen between the number of Gd enhanced MRIs and increase in SI ratio in the dentate nucleus (DN) and globus pallidus (GP). This effect was only statistically significant in the VHL group

vs. = versus

### Kidney and liver function

The kidney function of most VHL patients was normal, although the majority had renal cysts on imaging and some of them suffered from renal cell carcinomas. Of the 25 patients with a normal kidney function, 12 patients had Gd accumulation in the dentate nucleus and/or globus pallidus.

Accumulation in both regions was observed in 2 of 3 patients with chronic renal failure stage 2 (5 and 12 Gd enhanced MRIs), whereas a third patient with stage 2 (19 Gd enhanced MRIs) did not have accumulation. One of the VHL patients who underwent >20 Gd enhanced MRIs and a kidney transplant in the past, had accumulation in both regions. All VHL patients had a normal liver function.

Similarly, most TSC patients had a normal kidney function, although the majority suffered from renal cysts and angiomyolipomas. Of the 20 patients with a normal kidney function, 13 patients had accumulation in the dentate nucleus and/or globus pallidus.

One male patient with chronic renal failure stage 2 and >20 Gd enhanced MRIs had accumulation in both regions. Three patients had chronic renal failure stage 3.One of them was a 9-year old boy with polycystic kidney disease who had no accumulation after 8 Gd enhanced MRIs. The second patient was a 44-year old male with a left nephrectomy and multiple angiomyolipomas on the right kidney, who had accumulation after >20 Gd enhanced MRIs. The third one was a 68-year old female with a left nephrectomy and small cysts on the right kidney, who underwent >20 Gd enhanced MRIs, and had accumulation as well.

All TSC patients had a normal liver function.

## Discussion

Patients affected by hereditary tumor syndromes, such as VHL and TSC, undergo periodical Gd MRI screening, according to international guidelines [1, 2]. However, the accumulation of Gd has recently been described in diverse tissues, including the brain [6, 7].

It is unknown whether Gd accumulation is influenced by the type of underlying disease. To address this important question, we retrospectively analyzed the prevalence and rate of Gd accumulation in a comparative study of VHL and TSC patients.

We observed Gd accumulation in the dentate nucleus and globus pallidus in a very large subset of VHL patients after more than 5 Gd enhanced MRIs, and even before. The prevalence and rate of accumulation in the dentate nucleus and globus pallidus was lower in the TSC group.

To date, there are no other studies about individual variations in Gd accumulation. Kanda et al. [22] and McDonald et al. [21] reported Gd deposition in healthy patients with a normal blood-brain barrier, and thus, without correlation to disease status.

In both groups, a linear correlation was observed between number of Gd enhanced MRIs and increase in signal intensity in the dentate nucleus and globus pallidus. Whereas there was a linear positive trend in both groups, a statistically significant linear correlation was only present in the VHL group, not in the TSC group.

In general, the linear trend of accumulation over time is in concordance with other studies. Visible accumulation of Gd on T1-w MRI typically follows a linear increase starting from 5 to 6 Gd administrations. A recent systematic review by Olchowy et al. [23] reported a positive correlation between repeated administration of Gd and increased signal intensity of the dentate nucleus and globus pallidus on T1-w MRI, in 1247 patients from 25 different publications [24–26]. However, groups were almost consistently composed of patients suffering from different diseases and a distinction between the phenotypes of patients undergoing MRIs was never made. Our study demonstrates the presence of variable accumulation patterns in different subgroups of patients.

The observation that the linear increase of accumulation in our TSC patients was not statistically significant, may first of all be explained by the small study population. Second, Gd typically migrates through damaged blood-brain barriers. Because the majority of our TSC patients are children in late childhood or adolescence, their blood-brain barrier is more intact [27–29]. Therefore, accumulation may have happened more slowly than in older patients with a damaged blood-brain barrier due to their longer existing disease. Third, our imaging analysis program may not have been sensitive enough for the assessment of small linear increases in signal intensity.

A fourth hypothesis is that in the TSC group, as these patients are younger than our VHL patients, more of them may have received ionic linear GBCAs instead of non-ionic linear GBCAs. Ionic linear GBCAs were introduced more recently than non-ionic linear-GBCAs and typically accumulate more slowly [30].

A fifth reason for the difference in accumulation is that TSC patients have less tumor load compared to VHL patients, who typically develop multiple posterior fossa tumors and thus harbor severe blood-brain barrier disruption with microvascular leakage.

We conclude that some patients may have a predisposition for Gd accumulation, due to several potential individual factors such as age and degeneration of the brain-blood barrier, differences in tumor load, proximity of a tumor to the dentate nucleus and/or globus pallidus. Again, larger samples will be required to investigate these different hypotheses.

Due to the small subset of patients (*N* = 4) with severe renal impairment, the effect of kidney function on Gd accumulation could not be evaluated with certainty. As the patients with severe renal impairment had already undergone many years of screening, the number of MRIs may have been a possible confounder for the effect of kidney function on accumulation. In the VHL and TSC groups with normal kidney function, there were as many patients without Gd accumulation as patients with accumulation, unrelated to years of screening. Furthermore, some patients with an abnormal kidney function who underwent a large number of Gd enhanced MRIs, did not have accumulation. According to the literature, kidney and liver function do not significantly influence the accumulation of Gd in the central nervous system, because accumulation has been observed in patients with a normal kidney function as well [31, 32].

Possible limitations of our study include its retrospective nature and the fact that duration of screening was used instead of the exact amount of contrast-enhanced MRIs. In addition, some patients may have received two or more different subtypes of contrast agents. As with other orphan diseases, patients go for follow-up in different centers and all future research focused on rare hereditary tumor syndromes will encounter these problems. Differences in Gd accumulation between subgroups of patients have thus far not been evaluated. Despite the abovementioned issues, our data may therefore provide further guidance for future research, which can be further focused on the different subgroups of patients in whom Gd accumulation happens. Knowledge about the mechanism of accumulation and the subgroups of patients in which accumulation occurs more frequently, can contribute to research about the mechanisms and clinical impact of accumulation.

None of our patients experienced immediate or long term clinical side-effects of Gd toxicity. On the other hand, as VHL and TSC patients are prone to the development of several central nervous system tumors, causing a broad range of neurological symptoms, the attribution of neurological deterioration to long term Gd accumulation should be done with caution. However, there was no mention of any inexplicable symptoms in the patients’ files that could not be related to VHL or TSC disease progression.

## Conclusions

Our results demonstrate that the accumulation of Gd occurs in almost all patients who are regularly screened with Gd enhanced MRIs, especially after more than 5 MRIs. Gd accumulation in the dentate nucleus and globus pallidus is very frequently observed in VHL patients, being present in all patients after 16 MRIs. The observation that the prevalence of accumulation increases together with increasing numbers of MRIs is also made in TSC patients, but less pronounced. The higher prevalence and rate of accumulation in VHL patients may be explained by the typical vascular leakage accompanying central nervous system hemangioblastomas.

Neurological consequences of Gd accumulation remain unknown, neither can they be derived from the results of this study. However, because of the significant prevalence of Gd accumulation we have changed our screening protocol for VHL and TSC patients in order to minimize the amount of Gd injection. In the past, patients received an average of 2–3 Gd doses per year to separately screen for the brain, spine and abdomen. Now, only a single administration of any GBCA is used to simultaneously screen for brain, spine and/or abdominal lesions in an abbreviated MRI protocol focused specifically on either VHL or TSC lesions.

Unenhanced MRI is not sensitive enough for the diagnosis of the majority of VHL and TSC lesions, however, abdominal ultrasound can occasionally be used for the follow-up of cystic lesions. We also consider to lengthen screening intervals on an individual basis if there is no disease progression since the last screening, and if there are no lesions close to the limit of treatment. Furthermore, for VHL and TSC patients without central nervous system involvement, we suggest limiting MRI brain and spine screening to only once in 2 years.

## Abbreviations

eGFR: Estimated glomerular filtration rate
FWO-V: Fund for Scientific Research-Flanders
GBCAs: Gadolinium based contrast agents
Gd: Gadolinium
INR: International Normalized Ratio
MDRD: Modification of Diet in Renal disease
MRI: Magnetic resonance imaging
RCC: Renal cell carcinoma
ROI: Region of interest
T1/T2-w MRI: T1/T2-weighted magnetic resonance imaging
TSC: Tuberous Sclerosis Complex
VHL: Von Hippel-Lindau
